# HTLV-1 proviral load in infective dermatitis associated with HTLV-1 does not increase after the development of HTLV-1-associated myelopathy/tropical spastic paraparesis and does not decrease after IDH remission

**DOI:** 10.1371/journal.pntd.0007705

**Published:** 2019-12-18

**Authors:** Everton S. Batista, Pedro D. Oliveira, Janeusa Primo, Cinthya Maria Neves Varandas, Ana Paula Nunes, Achiléa L. Bittencourt, Lourdes Farre

**Affiliations:** 1 Laboratory of Experimental Pathology, Gonçalo Moniz Research Center, Oswaldo Cruz Foundation (CPQGM/FIOCRUZ), Salvador, Bahia, Brazil; 2 Department of Internal Medicine, Prof. Edgard Santos Teaching Hospital, Federal University of Bahia, Salvador, Bahia, Brazil; 3 Neuropediatric Department, Santo Antonio Hospital of Sister Dulce's Social Work, Salvador, Bahia, Brazil; 4 Department of Pathology, Prof. Edgard Santos Teaching Hospital, Federal University of Bahia, Salvador, Bahia, Brazil; 5 ProCURE Program, Catalan Institute of Oncology, IDIBELL, l’Hospitalet de Llobregat, Barcelona, Spain; National Institutes of Health, UNITED STATES

## Abstract

**Introduction:**

Infective dermatitis associated with HTLV-1 (IDH) is a recurrent eczema which affects children vertically infected with HTLV-1. In Bahia, Brazil, we recently reported that 47% of IDH patients also develop juvenile HTLV-1-associated myelopathy/tropical spastic paraparesis (HAM/TSP), a progressive disabling disorder which is typically reported in adult HTLV-1 carriers. IDH may also predispose to adult T-cell leukemia/lymphoma, a neoplasm associated with HTLV-1. The factors relating to the development of HTLV-1-associated juvenile diseases have not yet been defined. HTLV-1 proviral load (PVL) is one of the main parameters related to the development of HTLV-1 associated diseases in adults. In the current study, we investigated the role of PVL in IDH and juvenile HAM/TSP.

**Methodology/Principal findings:**

This is a cohort study that included fifty-nine HTLV-1 infected children and adolescents, comprising 16 asymptomatic carriers, 18 IDH patients, 20 patients with IDH and HAM/TSP (IDH/HAM/TSP) and five with HAM/TSP. These patients were followed-up for up to 14 years (median of 8 years). We found that PVL in IDH and IDH/HAM/TSP patients were similarly higher than PVL in juvenile asymptomatic carriers (p<0.0001). In those IDH patients who developed HAM/TSP during follow-up, PVL levels did not vary significantly. HAM/TSP development did not occur in those IDH patients who presented high levels of PVL. IDH remission was associated with an increase of PVL. Inter-individual differences in PVL were observed within all groups. However, intra-individual PVL did not fluctuate significantly during follow-up.

**Conclusions/Significance:**

High PVL in IDH patients was not necessary indicative of progression to HAM/TSP. PVL did not decrease after IDH remission. The maintenance of high PVL after remission could favor early development of ATL. Therefore, IDH patients would have to be followed-up even after remission of IDH and for a long period of time.

## Author summary

IDH is a recurrent eczema caused by the human T-cell lymphotropic virus type-1 (HTLV-1) that occurs mainly in children and adolescents. IDH disappears in adulthood but may predispose to the early development of other HTLV-1 associated diseases such as HTLV-1-associated myelopathy/tropical spastic paraparesis (HAM/TSP) and adult T-cell leukemia/lymphoma (ATL). In Bahia, Brazil, 47% of the IDH patients develop HAM/TSP. In this study, we evaluated HTLV-1 proviral load (PVL) in a cohort of children and adolescents with HTLV-1 infection who were followed-up during 14 years in the different clinical settings of IDH and HAM/TSP. IDH is considered a risk factor for HTLV-1 associated diseases in adults. We observed that PVLs in patients with IDH and in those with IDH and HAM/TSP were similar and, in both groups, higher than in asymptomatic carriers. Moreover, high PVL in these patients did not predispose necessarily to HAM/TSP. PVL remained high after IDH remission. Intra-individual PVL did not fluctuate significantly during the 14 years of follow-up. Our results indicate the importance of monitoring IDH patients even after remission because they remain with high levels of PVL that can favor the development of ATL.

## Introduction

HTLV-1 can cause severe diseases such as infective dermatitis associated with HTLV-1 (IDH), HTLV-1-associated myelopathy/tropical spastic paraparesis (HAM/TSP), and adult T-cell leukemia/lymphoma (ATL), among others. IDH is a chronic and severe form of exudative and infective childhood dermatitis. HAM/TSP is a chronic myelopathy characterized by slow progressing spastic paraparesis and ATL is an aggressive, mature T-cell malignancy characterized by a poor response to chemotherapy [1–3].

IDH occurs mainly during childhood and adolescence while ATL and HAM/TSP are considered diseases of adulthood. Juvenile HAM/TSP is rare, with only 27 cases reported [1]. Interestingly, it is frequently associated with IDH. Around 47% of the IDH patients in Bahia, Brazil develop HAM/TSP even in adolescence [2]. Juvenile HAM/TSP is clinically similar to adult HAM/TSP but is mainly associated with vertical HTLV-1 transmission and can present rapid progression [2]. The factors predisposing to juvenile HAM/TSP in IDH patients were not yet established. In adults, the HTLV-1 proviral load (PVL) has been extensively studied as one of the main risk factors for the development and progression of the HTLV-1-associated diseases, with a special relevance in HAM/TSP [3]. In the setting of early-onset HTLV-1 related diseases, this parameter has not yet been properly investigated. We previously reported that IDH patients showed higher PVL compared to adult asymptomatic carriers, and similar to adult HAM/TSP patients [4]. In that previous study, it was suggested that high levels of PVL in IDH may be related to the high frequency of HAM/TSP in these patients. However, until now it was not assessed whether PVL could be a good marker for monitoring early HAM/TSP development in IDH patients, namely, if IDH patients with high PVL are those with high predisposition to develop this myelopathy.

IDH may also predispose to ATL. Five cases of early onset ATL in IDH patients have been reported, three cases having originated in Bahia [1]. Moreover, 37.5% of the ATL cases in Bahia reported a history of severe eczema in childhood suggestive of IDH [5].

IDH disappears when patients reach puberty [6]. It is unknown if IDH remission is accompanied by a decrease in the numerous circulating infected cells detected during active disease [4].

Our hypothesis were: (i) In IDH patients PVL increases after the HAM/TSP development (ii) HAM/TSP development occurs in those IDH patients with higher PVL; (iii) PVL decreases after IDH remission.

The aim of the current study was to investigate PVL in early onset HTLV-1 diseases with special focus on the association of HAM/TSP with IDH and on IDH remission. Patients were followed-up during 14 years and inter-individual and intra-individual PVL variations in this period were evaluated in the setting of disease progression.

## Methods

### Study population and samples

This is a cohort study that included fifty-nine children and adolescents HTLV-1 infected, all from the State of Bahia, Brazil. Sixteen were asymptomatic carriers (asymptomatic group), 18 had IDH (IDH group), 20 had IDH and HAM/TSP (IDH/HAM/TSP group) and five had HAM/TSP (HAM/TSP group). They were diagnosed and followed-up at the dermatology outpatient clinic of the Professor Edgard Santos Teaching Hospital, Federal University of Bahia, between 2002 and 2017. This was a convenience sample and we included all IDH patients, those with HAM/TSP association, HAM/TSP patients and asymptomatic carriers attended in the outpatient clinic during the mentioned period. Asymptomatic carriers and HAM/TSP patients were siblings or relatives of the IDH patients or cases derived from other clinical services of the State of Bahia. The dermatology outpatient clinic of the Professor Edgard Santos Teaching Hospital is the reference ambulatory of juvenile HTLV-1 infection in the State of Bahia. All of them were HTLV-1-positive (ELISA confirmed by Western Blot or PCR) and HIV-negative. IDH and HAM/TSP diagnoses were performed according to previously established criteria [6–8]. Most of the patients were examined at least once a year, however in some cases this period was longer due to the socio-economic difficulties of this population. At each visit, neurological and dermatological examinations were performed including measurement of Osame Motor Disability Score (OMDS) [9] and Expanded Disability Status Score (EDSS) [10] and the participants were submitted to routine laboratorial exams. Dermatological aspects of these patients were previously described [6], while neurological aspects of HAM/TSP patients were recently reported [2]. IDH patients with active disease were treated with systemic sulfamethoxazole-trimethoprim. Patients who had had no treatment for a period of > 6 months and who presented no signs of the skin disease were considered to be in remission of IDH [6]. Relevant demographic and clinical data plus follow-up period of these groups were described in Table 1.

**Table 1:** Demographic and clinical data plus follow-up period of the studied groups.

	Asymptomatic carriers	IDH group	IDH/HAM/TSP group	HAM/TSP group
	n = 16	n = 18	n = 20	n = 5
Age^#^	12.50 (4–18)	14,00 (3–18)	13.50 (2–18)	14.00 (12–18)
Males/females	8/8	9/9	4/16	1/4
Age at IDH onset^#^	-	2 (1m-16)	2 (5m -15)	-
Cases with IDH remission	-	6	8	-
Age at HAM/TSP onset^#^	-	-	12.5 (4–20)	17 (11–18)
Total follow-up^#^	5.5 (2–8)	6 (3–14)	8 (3–14)	3 (3–10)

^#^values expressed in median (years) and range; m- month (when indicated).

Peripheral blood samples (15ml in EDTA) for each participant were collected at diagnosis and during follow-up. Peripheral blood mononuclear cells (PBMC) were separated by density gradient centrifugation using Histopaque 1077 (Sigma Aldrich). DNA was extracted using a QIAamp DNA blood Mini kit according to the manufacturer’s instructions (QIAGEN, Germany).

### PVL quantification

HTLV-1 PVL was quantified by real-time PCR using 30 ng of DNA and the *TaqMan Fast Universal PCR Master Mix* kit (Applied Biosystems) in the *7500 Fast Real-Time PCR System* (Applied Biosystems). The sequences of the primers and probes were as follows: 5’-CCC ACT TCC CAG GGT TTG GA-3’, 5’-GGC CAG TAG GGC GTG A-3’, and 5’-FAM/CCA GTC TAC/ZEN/GTG TTT GGA GAC TGT GTA CA/3IABkFQ/-3’for the *tax* gene and 5’- GTG CAC CTG ACT CCT GAG GAG A-3’, 5’-CCT TGA TAC CAA CCTGCC CAG-3’and 5’-FAM/AAG GTG AAC/ZEN/GTG GAT GAA GTT GGT GG/3IABkFQ/-3’for the *beta-globin* gene. The reaction conditions were 95ºC for 20s and then 40 cycles of 3s at 95ºC, followed by 30s at 60ºC.

PVL was quantified as the number of HTLV-1 DNA copies per 100 PBMCs, was calculated according to the following formula: = (copy number of Tax)/ (copy number of beta-globin/2)]·100, and was expressed HTLV-1 copies per 100 PBMC or as percentage.

### Statistical analysis

The GraphPad Prism5.02was used to analyze and plot patients’ data. Demographic and PVL data were expressed in median with range. The Mann-Whitney U test was used to compare data between two independent groups and was corrected by Bonferroni test. Wilcoxon matched-pairs signed rank test was used to compare paired groups. Correlations were examined using Spearman’s rank correlation coefficient. P-values < 0.05 were considered statistically significant.

### Ethics statement

The Research Ethics Committee of the Professor Edgard Santos Teaching Hospital of the Federal University of Bahia, Brazil approved this study protocol (number 3.002408). The parents or legal guardians of the participants gave written informed consent.

## Results

### PVL in early onset HTLV-1 diseases

**Fig 1:**
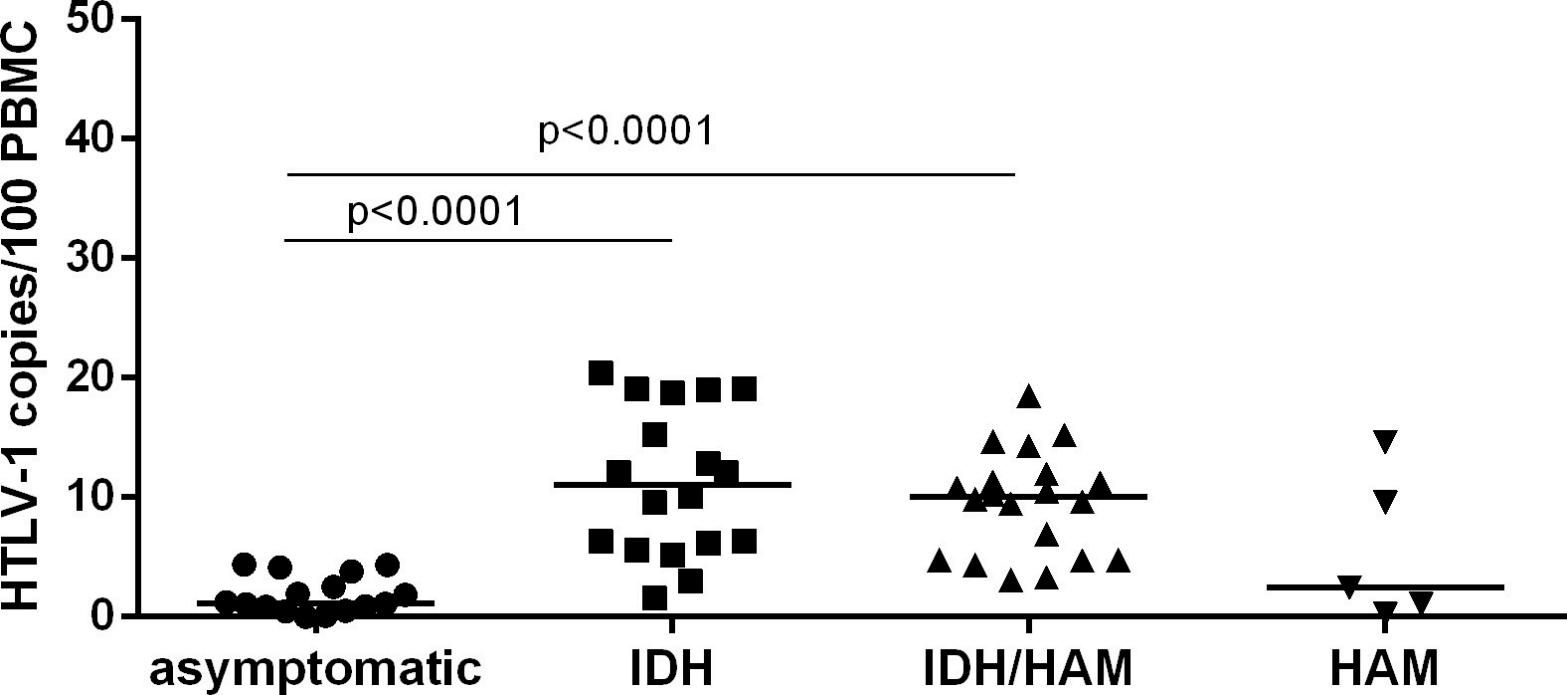
PVL in juvenile HTLV-1 associated diseases. Comparison of PVL between asymptomatic carriers, and IDH, IDH/HAM/TSP and HAM/TSP patients (Mann-Whitney U test, corrected by Bonferroni test).

IDH patients showed higher PVL than asymptomatic carriers, with median of 11.08 and 1.16 HTLV-1 copies/100 PBMC, respectively (p<0.0001) (Fig 1). Patients with IDH/HAM/TSP showed a PVL of 10.03 HTLV-1 copies/100 PBMC, similar to PVL in IDH patients (p = 0.4737) and higher than in asymptomatic carriers (p<0.0001). HAM/TSP patients without IDH showed a median PVL of 2.45 HTLV-1 copies/100 PBMC, similar to asymptomatic carriers. Although PVL in the HAM/TSP group was four times lower than PVL in IDH and IDH/HAM/TSP groups, this difference was not statistically significant.

### PVL remained similar in IDH patients after HAM/TSP development

**Fig 2:**
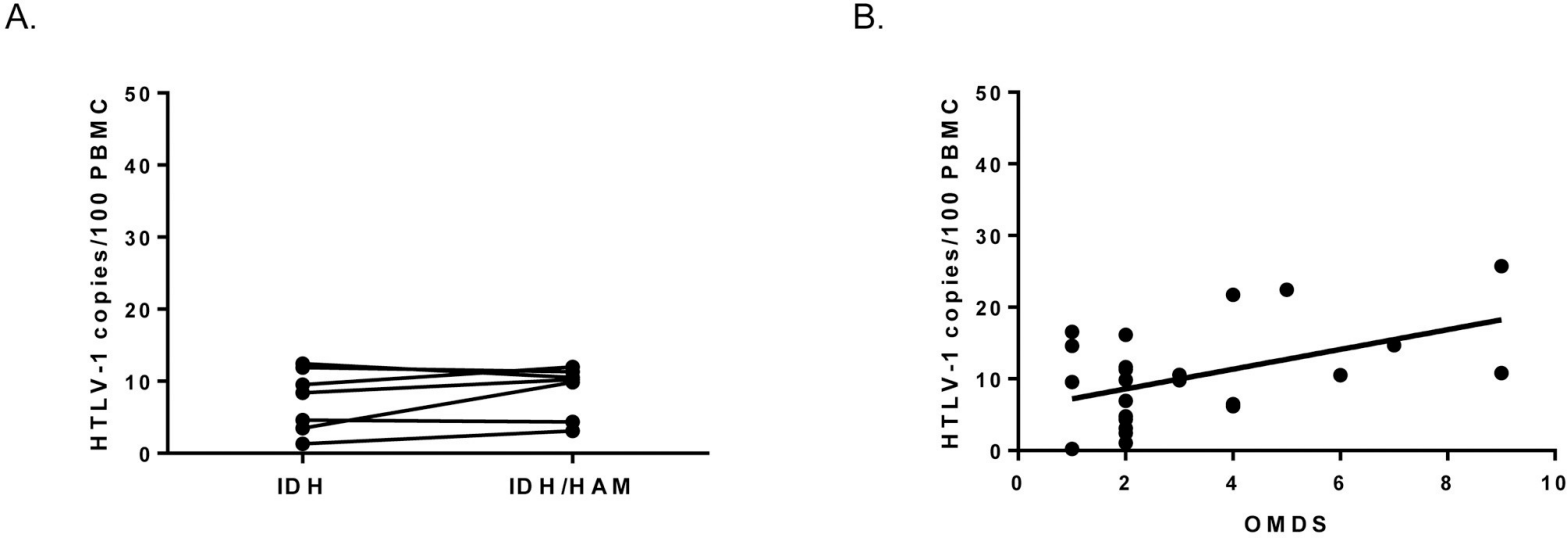
PVL in relation to HAM/TSP development. (A) Evolution of PVL in IDH patients before and after HAM/TSP appearance (Wilcoxon matched-pairs signed rank test, p = 0.3650). (B) Correlation between PVL and final OMDS at the end of follow-up (p = 0.0332, r = 0.4775, Spearman’s rank correlation coefficient).

Seven IDH patients developed HAM/TSP during follow-up. For these patients, median period of time between IDH diagnosis and HAM/TSP diagnosis was 7 years (range from 3 to 10 years). For each patient, it was possible to compare PVL before and after HAM/TSP development (intra-individually, paired samples) and they were similar, no significant differences were observed (p = 0.3650, Wilcoxon matched-pairs signed rank test) (Fig 2A). It is important to note that IDH preceded HAM/TSP manifestation in all IDH/HAM/TSP patients [2].

### Higher levels of PVL in IDH patients were not related to HAM/TSP development

To assess whether IDH patients who developed HAM/TSP were those with high PVL within IDH group, PVL of IDH patients before developing HAM/TSP were compared with PVL of IDH patients who did not develop HAM/TSP. No significant differences were observed (8.41 and 11.08 HTLV-1 copies/100 PBMCs, respectively, p = 0.0967, Mann-Whitney U test). Moreover, we checked these findings also stratifying IDH patients by PVL (≤ than 10 copies/100 PBMC and > than 10 copies/100 PBMC). In the IDH group who did not develop myelopathy, 55% (10 in 18) of patients presented more than 10 copies/100 PBMC. However, in IDH group who developed HAM/TSP, only 28% (2 in 7) presented more than 10 copies/100 PBMC.

Final OMDS and EDSS values for HAM/TSP patients at the end of follow-up ranged from 1 to 9 and from 1 to 10, respectively. A weak but significant positive correlation was observed between PVL and final OMDS (p = 0.0332, r = 0.4775) (Fig 2B). No association was observed between PVL and EDSS.

### IDH remission is associated with an increase in PVL

**Fig 3:**
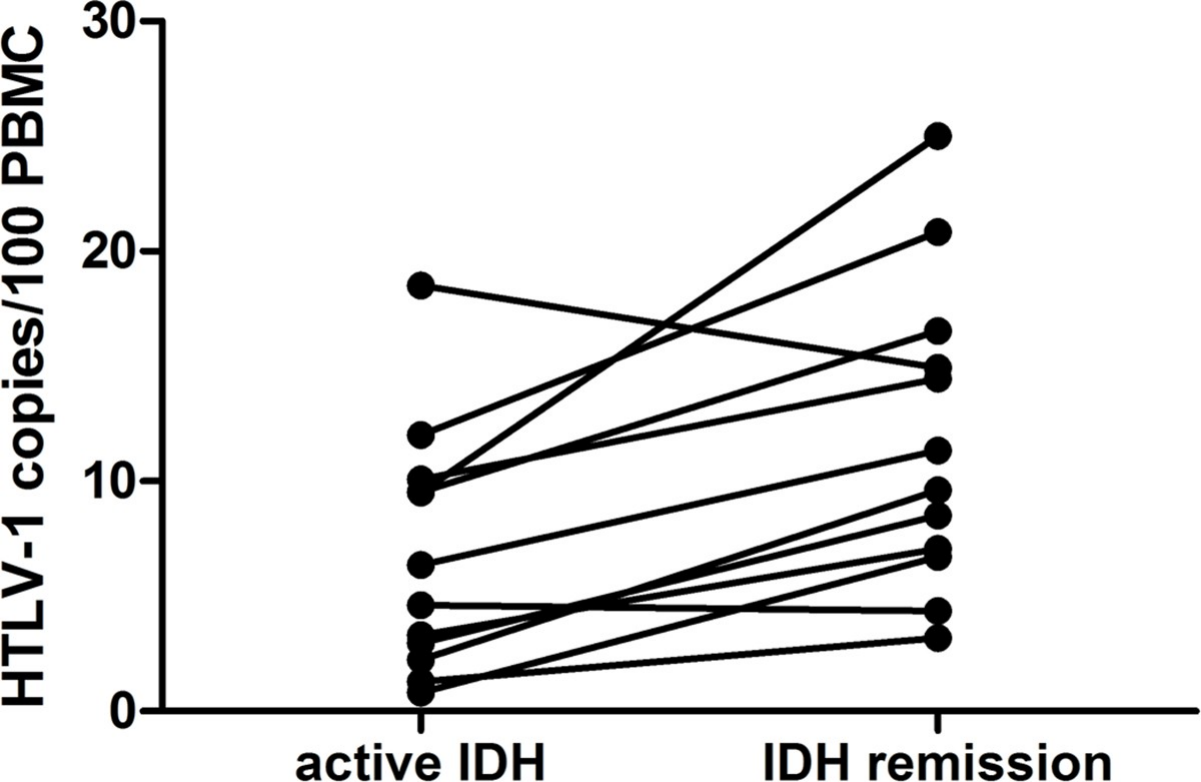
PVL at IDH remission. Comparison of PVL in patients with active IDH and after IDH remission (p = 0.0034, paired samples, Wilcoxon matched-pairs signed rank test). Remission of IDH was considered when a patient off treatment remained free of disease for at least six months.

Fourteen patients presented IDH remission during the follow-up period (Table 1). Median age at remission was 15 years old (range 11 to 26 years old). In 12 of them, it was possible to compare PVL in active IDH and after remission. PVL was higher in IDH remission than in active IDH for paired samples (p = 0.0034, Wilcoxon matched-pairs signed rank test) (Fig 3).

### Evolution of PVL

As HTLV-1 infection in these children and adolescents occurred vertically, by breastfeeding[2,6], we tested whether the levels of PVL correlated with age. PVL did not correlate with age when considering all individuals included independently of clinical conditions (p = 0.2725, r = -0.1733, Spearman’s rank correlation) or within disease groups.

**Fig 4:**
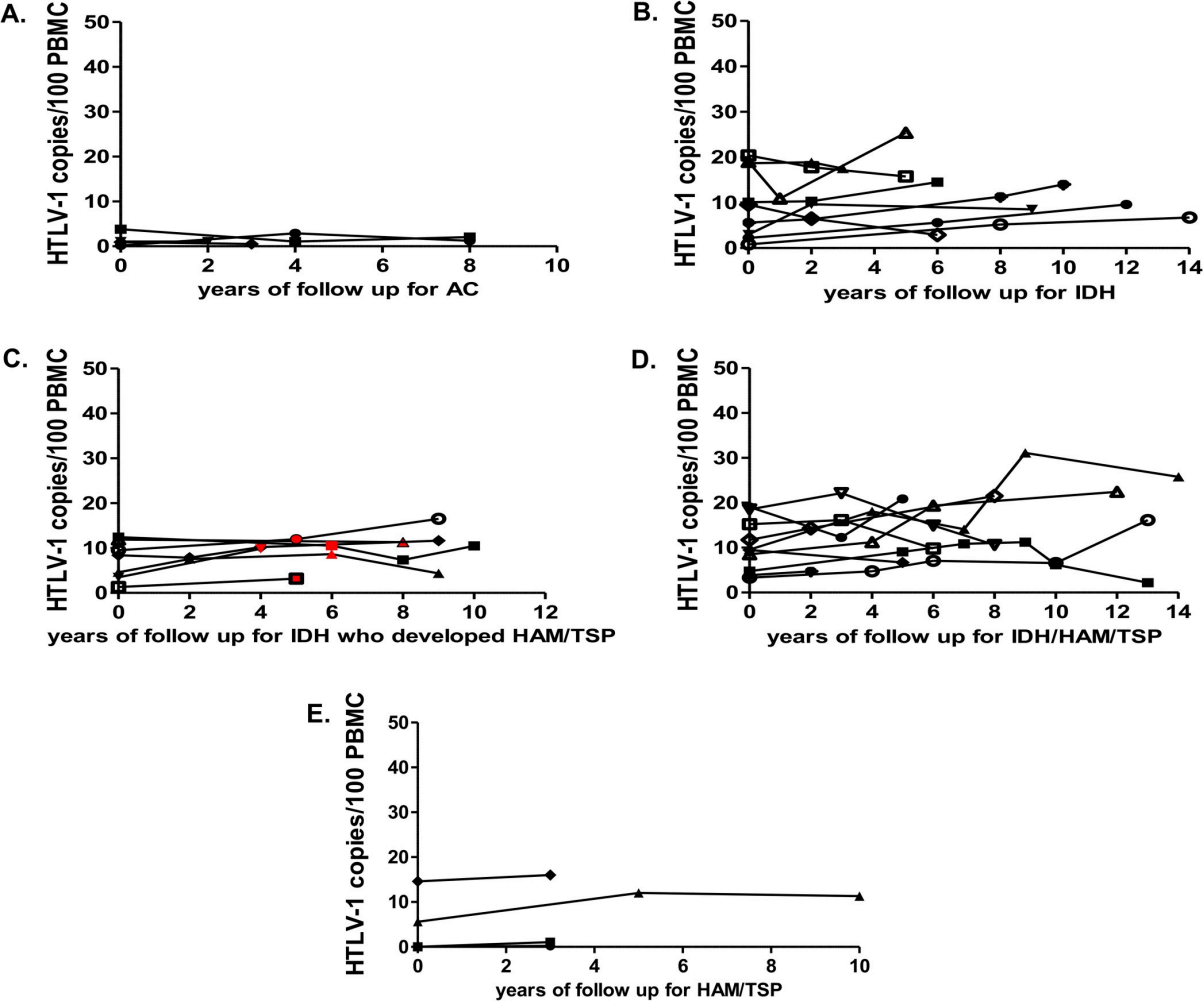
Evolution of PVL during follow-up in different clinical settings. (A) PVL during follow-up (in years) since investigation of HTLV-1 serology for asymptomatic carriers; (B) PVL during follow-up (in years) since diagnosis for IDH patients; (C) PVL during follow up (in years) since IDH diagnosis for IDH patients who developed HAM/TSP during follow-up (the red point in the plot indicate the occurrence of HAM/TSP diagnosis); (D) PVL during follow up (in years) since diagnosis of both IDH and HAM/TSP for those patients with both diseases; (E) PVL during follow-up (in years) since HAM/TSP diagnosis for patients with HAM/TSP without IDH.

For the analysis of intra-individual PVL evolution, we included the participants who had at least two measures of PVL during a follow-up period of no less than three years (up to fourteen years). They were: (i) four asymptomatic carriers (Fig 4A); (ii) 11 IDH patients (Fig 4B); (iii) the seven IDH patients who developed HAM/TSP during follow-up (Fig 4C); (iv) 11 patients who had simultaneous diagnosis of IDH and HAM/TSP (Fig 4D); (v) 3 HAM/TSP patients (Fig 4E). Intra-individual PVL levels presented a moderate fluctuation during follow-up.

**Fig 5:**
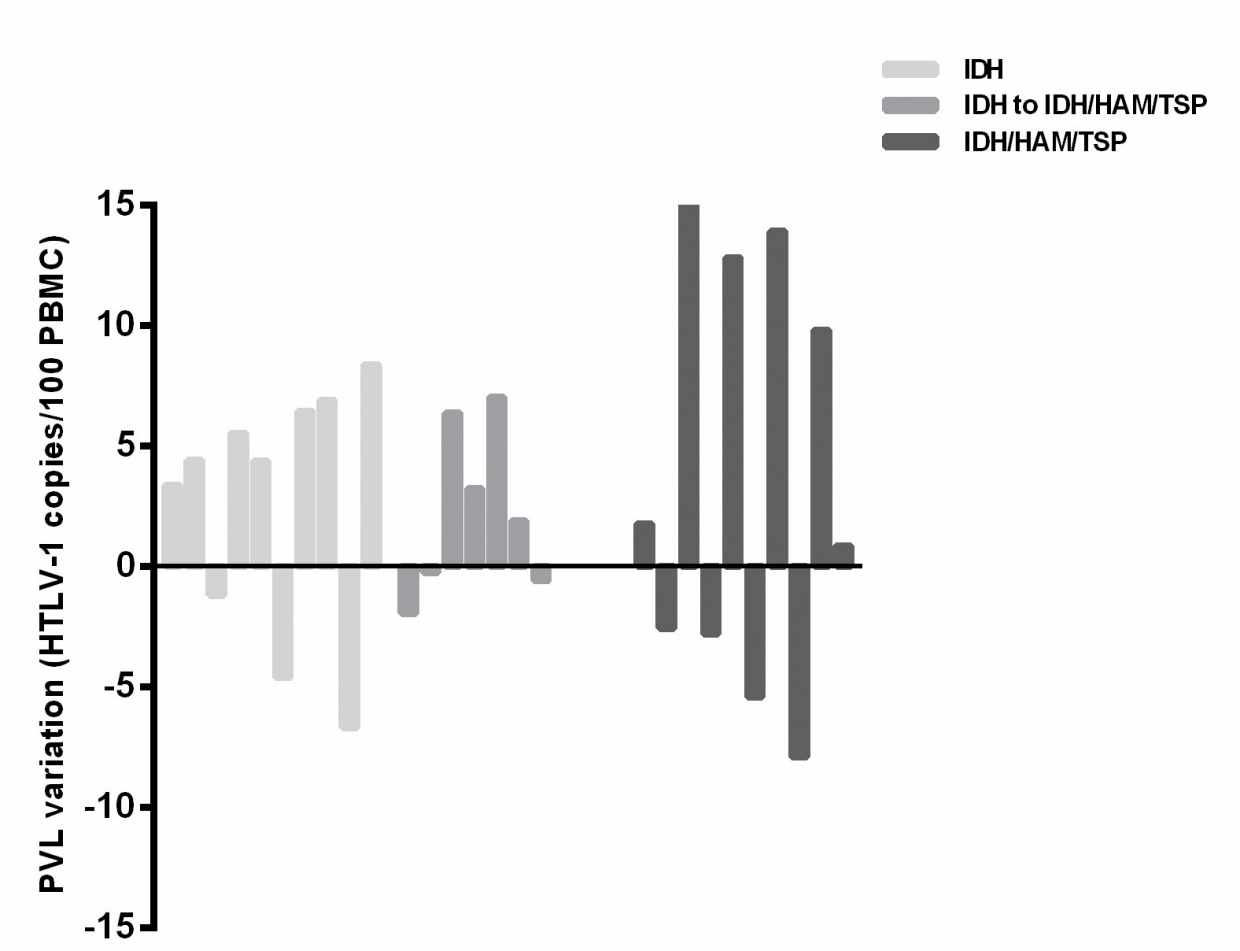
Variations between initial and final PVL values. Variation between initial and final PVL (at the end of follow up) values in a period of least five years for IDH, IDH patients who developed HAM/TSP during follow-up and IDH/HAM/TSP patients. Values were calculated according to the following formula: variation = final PVL—initial PVL, and were expressed as HTLV-1 copies/100 PBMC.

Variations between initial and final PVL values in a period of at least five years were shown in Fig 5.

As PVL did not correlate with age and it remained without significant fluctuations during follow up, we then evaluated whether earlier onset of HTLV-1 diseases were associated with higher PVL. Levels of PVL also did not correlate with age at IDH onset (p = 0.0619, r = -0.4433, Spearman’s rank correlation) or age at HAM/TSP onset (p = 0.0770, r = -0.3600, Spearman’s rank correlation). Moreover, age at onset of neurological manifestations were compared between HAM/TSP patients (with or without IDH) with ≤ than 10 copies/100 PBMC (n = 12) and with > than 10 copies/100 PBMC (n = 13) no statistical differences were observed (p = 0.1228, Mann-Whitney U test). Patients with ≤10 copies presented a median age at neurological manifestations of 12 years old (range 3 to 18 years) while for patients with >10 copies it was 8 years old (range 5 to 17 years).

Four pairs of siblings were found among the patients. In two of the four pairs, both siblings had IDH/HAM/TSP. In the two other pairs, one sibling had IDH/HAM/TSP and the other had only HAM/TSP. In the first pair, whose members were both IDH/HAM/TSP patients PVLs f were 11.23 for one sibling and 7.06 HTLV-1 copies/100 PBMC for the other). In the second pair, PVLs were 15.24 for one sibling and 11.23 HTLV-1 copies/100 PBMC for the other. In the other two pairs of siblings with different clinical status, IDH/HAM/TSP patients of each pair presented higher PVLs than their respective HAM/TSP siblings (10.8 vs 2.45 for one pair and 3.12 vs 1.07 HTLV-1 copies/100 PBMC for the other).

## Discussion

In this study, we demonstrated that the high levels of PVL observed in IDH patients remained similar after the development of juvenile HAM/TSP and did not decrease after IDH remission.

In the literature, there are almost no data on PVL in children and adolescents with HTLV-1. Our group previously reported PVL in IDH in comparison with adult asymptomatic carriers and adult HAM/TSP patients [4]. Here we studied PVL in different clinical settings in childhood and adolescence. Besides IDH patients, we included juvenile asymptomatic carriers, IDH patients who developed HAM/TSP (some of them during follow up) and a small group of juvenile HAM/TSP.

PVL in our study did not exceed 25% of infected cells in the PBMC in any clinical condition. IDH patients presented higher PVL than asymptomatic carriers of the same age range, as observed when compared with adult asymptomatic carriers [4]. Indeed, juvenile asymptomatic carriers presented PVL levels similar to adult asymptomatic carriers [11].

Considering that HAM/TSP in adults is characterized by high PVL [11], we expected that the association of HAM/TSP with IDH would be accompanied by an increase in PVL. However, we observed similar PVL in IDH patients with or without myelopathy. Even though, no increase was observed in PVL intra-individual values in those IDH patients who developed HAM/TSP during follow-up. Moreover, those IDH patients who developed HAM/TSP were not those who presented higher levels of PVL, suggesting that high levels of PVL in IDH patients may favor but do not necessarily trigger the development of myelopathy. Other parameters would have to be investigated to understand why IDH patients developed HAM/TSP much earlier and more frequently (47% of IDH patients in Bahia State, Brazil) [2] than adult asymptomatic carriers (frequency estimated to be around 3%) [12]. One possibility would be the immunological deregulation that occurs in IDH that is similar to that observed in HAM/TSP [12]. Moreover, genetic factors could also predispose to this myelopathy, as reported for human leukocyte antigen (HLA) and other gene polymorphisms [13–16]. Therefore, high PVL in IDH patients was not indicative of progression to HAM/TSP.

We previously referred that IDH patients had PVL similar to that of adult HAM/TSP [4]. In the current study, median PVL in juvenile HAM/TSP patients was four fold lower than PVL in IDH patients. However, no statistically significant differences were observed between these groups probably due to the small number of patients included in the juvenile HAM/TSP group. The small number of individuals included in our study constitutes one of the main limitations of it. In Bahia, as well as in other endemic regions in developing countries, the majority of HTLV-1 carriers are unaware of their infection. Usually, the infection is investigated only when some case of associated disease occurs in the family. This makes it difficult to identify juvenile asymptomatic carriers. On the other hand, the IDH develops in a very small percentage of HTLV-1 carriers. Juvenile HAM/TSP is also rare, especially when not associated with IDH Therefore, these results raise the question of whether juvenile HAM/TSP has lower PVL levels than adulthood HAM/TSP and if high PVL could become a risk factor for the development of juvenile HAM/TSP as considered in adult HAM/TSP [3]. Three of the five juvenile HAM/TSP patients had < 2.5% of infected cells in PMBC without significant variations during three years (even presenting OMDS and EDSS between 2 and 3). While, in IDH/HAM/TSP group the smallest PVL value was 3% of infected cells in PBMC. One possibility is that IDH more than HAM/TSP determines the PVL levels.

During 14 years of follow-up, intra-individual PVL fluctuations in IDH and IDH/HAM/TSP groups were discrete as occurs in adult carriers [17]. In the IDH/HAM/TSP group, one case presented higher variation: an increase from 9 to 25 copies/100 PBMCs during 14 years of follow-up with rapid evolution of myelopathy (with initial and final OMDS of 4 and 8, respectively) [2]. Increase of PVL in myelopathy cases with rapid evolution was also reported in adults [3].The levels of PVL in IDH remission setting were also unexpected. IDH patients in remission had higher PVL than when the disease was active. IDH remission became definitive without relapses. Notwithstanding, IDH patients even without relapses remained with high PVL. In IDH remission, that occurred spontaneously in the beginning of adulthood, the use of antibiotic therapy is no more necessary because the associated secondary bacterial infection is absent. Considering our data, we may hypothesize that IDH remission may be related to an acquired capacity to control the secondary bacterial infection more than a reduction of the PVL. Considering that in Bahia, around 37% of the ATL patients with cutaneous involvement have a history compatible with IDH in childhood [18], the maintenance of a high PVL may favor the appearance of a predominant clone with high proliferative capacity and subsequent development of ATL [19].

Our results suggest that IDH patients must be clinically followed-up even after remission and for a long period.

## Supporting information

S1 Checklist STROBE checklist. (PDF)
